# NAVIGATING PAID CAREGIVING IN HOSPICE DURING COVID-19: AN INTEGRATED MULTIPLE METHODS ANALYSIS

**DOI:** 10.1093/geroni/igae098.0393

**Published:** 2024-12-31

**Authors:** Patricia Kim, Emily Franzosa, Jonelle Boafo, Margaret McDonald, Laura Moreines, Daniel David, Melissa Aldridge

**Affiliations:** Icahn School of Medicine at Mount Sinai, New York, New York, United States; James J Peters VA Medical Center, Bronx, New York, United States; Icahn School of Medicine at Mount Sinai, New York, New York, United States; Visiting Nurse Service of New York, New York, New York, United States; New York University, New York, New York, United States; NYU College of Nursing, New York, New York, United States; Icahn School of Medicine at Mount Sinai, New York, New York, United States

## Abstract

Paid caregivers are integral members of the end-of-life care team, but navigating paid care services is complex. Families must often work across multiple providers including hospice, home health, private pay, and government to coordinate the care they need to help patients die at home. We examined experiences with end-of-life paid caregiving among 61 patients in a large, New York City health system during the first year of the COVID-19 pandemic. We analyzed care trajectories of patients who enrolled in hospice and died between March 1, 2020-March 31, 2021, integrating data from real-time linked medical and hospice electronic health records with reflective interviews with 30 interdisciplinary team members at 3 organizations providing end-of-life care during the pandemic. We found: 1) Paid caregivers were the “eyes and ears” of the hospice team and paid caregivers’ work helped families keep patients at home; 2) Patients’ increasing end-of-life needs required additional paid caregivers, heightening patients’ infection risk; 3) Conflicting organizational policies across providers and payers, such as screening, vaccination, and visitation restrictions, disrupted aide care; and 4) Scope of practice restrictions for some types of paid caregivers reduced their ability to help. Our findings highlight both the essential support role of paid caregiving in end-of-life care, and regulatory/policy burdens that may reduce the benefit of paid caregiving at this critical juncture. Supporting stability and continuity in caregiving teams and building more flexibility into paid caregivers’ scope of practice and patients’ care plans may assist aides and families in managing end-of-life care.

